# “You’re Not Dealing with What We’re Dealing with”: COVID-19 Hesitancies and Motivations Among Late Vaccinating Black Americans in the Deep South

**DOI:** 10.1007/s40615-025-02479-6

**Published:** 2025-05-29

**Authors:** Anthony J. Stone, Madeline Arriaza, Claire Brindley, Elliot Meador, Wesley L. James, Karen Matthews

**Affiliations:** 1 Department of Sociology, University of Memphis, 231 Clement Hall, Memphis, TN 38152 USA; 2 Delta Health Alliance, 435 Stoneville Rd, Leland, MS 38756 USA; 3 Center for Community Outreach and Evaluation, University of Memphis, 221 Clement Hall, Memphis, TN 38152 USA

**Keywords:** COVID-19, Black Mississippians, Rural, Vaccine motivators, Vaccine hesitancy, Mississippi Delta

## Abstract

**Introduction and Background:**

The COVID-19 vaccines have been public for several years and have been shown to mitigate symptoms in recipients. Yet many Americans are still hesitant or late to receive the vaccines for several reasons, especially Black Americans. To better understand how Black Americans who reside in the rural Mississippi Delta understand the COVID-19 virus and vaccines, we conducted a qualitative study focusing on their hesitance toward the vaccine, and how they make decisions or change(d) their minds regarding receiving it at intersecting layers of social disadvantage—race and place-based inequalities.

**Methods:**

In 2021, we conducted 10 in-person, in-depth, semi-structured interviews with Black residents of the Mississippi Delta who were vaccinated months after the vaccines’ release. We used an iterative, grounded approach to study design and data analysis, as well as the constant comparative method, until data saturation was achieved.

**Results:**

Participants reported they were initially hesitant toward the vaccines for the following reasons: an overabundance of misinformation, fear of illness, mistrust of decision-making institutions, and uncertainty of unknown side effects. Participants cited several reasons that motivated them to change their minds and receive the vaccine, including positive results when people in their networks vaccinated, increased feelings of safety, work requirements, and faith in God.

**Conclusions:**

Our findings unpack the complications around vaccine hesitance and motivations for vaccine uptake for late vaccinators at the intersections of social and racial inequality. Findings underscore the importance of recognizing the pervasive influence of generalized institutional mistrust and spirituality when providing health advice to Black Mississippians about the COVID-19 vaccines.

## Introduction

The impact of the COVID-19 virus and the results of the subsequent pandemic have been a burden to individuals and communities across the world for nearly half a decade. Although everyone is exposed to the COVID-19 virus to some degree, the burden has been unevenly distributed across populations. In the USA, the virus exacerbates existing social inequalities in health across several key demographics, as evidenced by high rates of hospitalization and death among disadvantaged groups. One such demographic is race, where one-third of Black Americans are at high risk of becoming very ill with COVID-19 compared to roughly one-quarter of white Americans [1]. In many cases, Black folks are undertreated compared to whites, contributing to the gap between disparity and equity in COVID care received [2]. Relatedly, Black Americans account for a greater share of deaths and are three times as likely to die of COVID-19 compared with whites [2, 3]. Only Indigenous Americans (42%) are at higher risk than Black Americans, who are four times as likely to die compared to whites. These unequal health outcomes can be attributed to disparate access to care, restrictive lifestyle choices, as well as environmental, social, and economic inequalities [1, 4]. Additionally, constrained life choices (e.g., access to healthy foods and medicine, education, employment), disparities, and inequalities can be attributed as manifestations of racism rather than merely statistical differences [5]. Khazanchi and colleagues (2020) argue that the disproportionate number of minorities to enter the hospital due to COVID-19 compared to whites, and a subsequent lack of positive, proportional outcomes for minorities once admitted to the hospital supports their theory that “racism, not race” is to blame for the current inequities in health outcomes [6]. Said differently, although minorities enter hospitals for COVID-19 at higher rates, their positive health outcomes are fewer than whites. Moreover, the aforementioned disparities in health outcomes, inequalities in access to care, strained life choices, and the lack of statistical significance in achieved outcomes explicitly impact patient hesitancy to seek COVID-19 care or vaccination [7].

Race-based inequities are further exacerbated by place-based disparities. People living in rural areas of the USA face unique social and economic challenges that can make dealing with the virus’ impact more difficult than coping is for their urban-dwelling counterparts [8]. Rural areas have consistently higher poverty rates than metro areas [8], which are compounded by restricted job opportunities [9, 10]. The rural economy is primarily dominated by agriculture, forestry, tourism, and transportation. Jobs in these sectors are primarily seasonal, and many are informal—meaning that they do not offer an associated health insurance plan [11]. Furthermore, people living in rural areas are often geographically isolated and have difficulties accessing health care services, lack reliable high-speed broadband access for tele-appointments, and report high levels of stigma associated with care, especially mental health services [12]. All these factors were exacerbated during the COVID-19 pandemic in rural places.

There is clear evidence that the COVID-19 pandemic and subsequent lockdowns have had a disproportionate impact on both minority populations and those living in rural areas of the USA. The economic shutdown and subsequent reopening policies fueled an unemployment rate of about 9.74% in rural areas, compared to 7.40% nationally [13]. Indeed, evidence also supports that minority populations living in rural areas experience compounding effects [15] and are at an even greater risk of ill effects of the virus—along with the social and economic disruptions caused by its impacts. Moreover, such compounding factors are not dispersed evenly across racial groups in rural areas, and there is evidence that racial disparities in premature COVID-19 related death in rural areas disproportionally impact Black and Indigenous populations [14]. As such, the resulting COVID-19 vaccinations should offer a solution; however, minority populations remain hesitant to such a resolution.

COVID-19 vaccines were introduced in late 2020 to reduce the impacts and risks associated with both encountering and contracting the virus. However, since the rollout of the vaccines, vaccine hesitancy has remained a serious public health issue. Studies conducted before the release of the vaccine indicated that one-third of Americans were unsure about receiving the vaccine [16]. Basic reasons for this vaccine hesitancy across the USA include misinformation, lack of institutional trust, and apathy about COVID-19 [17]. For instance, Carpenter and colleagues (2024) found that from summer to fall 2020, vaccine hesitance among Black people increased (spiking in September) due to mistrust of the Food and Drug Administration and the Trump Presidential Administration. Notably, while hesitance for other groups leveled out around election time (October or November), Black folks’ hesitance was less likely to decrease and instead persisted [18]. Other research notes that hesitance may be moderated by place as well.

Recent research from residents in Arkansas suggests that vaccine hesitancy may be highest in non-Hispanic Black residents due to fear and general mistrust [19]. Similar research denotes that the combined effects of perceived discrimination and medical mistrust mediate racial minorities’ vaccine behavior [7, 19]. Morgan and colleagues (2023) found that medical mistrust and perceived discrimination decreased racialized minorities’ likelihood to become vaccinated. For Black Americans, the decision to vaccinate is not simply related to medical or institutional mistrust—it is complicated by evolving and diverging information about the vaccine, their concerns about vaccine integrity and risk, and social factors such as work mandates and familial responsibilities [20, 21]. Moreover, Black folks’ hesitancy is related to overall institutional mistrust in the government and health care systems thanks to systemic racism, (past) experimentation, and the limited number of Black elected officials in key roles regarding policy decisions and changes that impact Black Americans across issues [7, 19]. As such, vaccine hesitancy has remained consistent across at-risk demographics.

People have largely remained in their ideological camps regarding their motivations or decisions to get vaccinated or not. For example, in July 2021, 92% of those who said they would get vaccinated were vaccinated, and only 24% of those who said they would only get vaccinated if required have been vaccinated [21]. Although there is a general understanding about the reasons for vaccine hesitancy on a large scale, the differences in vaccine hesitancy are not well understood in populations that deal with intersecting layers of social disadvantage [22]. Moreover, many studies examining COVID-19 fear, care, vaccine hesitance, and motivations toward preventions rely on quantitative measures to determine who experiences such issues. Ignoring qualitative narratives means we know less about the “why” behind people’s hesitance or motivation to get vaccinated, for example. In the few existing qualitative studies, scholars have largely focused on reasons for refusing the vaccine [22] rather than the reasons for changing one’s mind [7]. Such a blind spot leaves out the hesitant or late vaccinators’ experiences or motivations to become vaccinated. This group could be crucial to informing vaccine education and outreach [23–25]. Moreover, understanding the experiences of at-risk populations in vulnerable, hard-hit regions might inform how to alter positive health motivation and vaccine uptake nationally.

In particular, one hard-hit rural region of the country is the Mississippi Delta, colloquially known as “The Delta.” This region is a 200-mile alluvial floodplain along the Mississippi River and is one of the most unique rural regions in the country. Its population is largely poor and Black, which is particularly distinctive for rural places in the USA. The region has a long history of social, economic, racial, and health inequality. However, it is more recently the site of an innovative intervention program to reduce the burden of COVID-19 on an already vulnerable population [15]. The Mississippi Recognizing Important Vaccine & Education Resources (RIVER) is a collaborative project between Delta Health Alliance (DHA), a nonprofit organization in the Mississippi Delta, the Town of Inverness, the Center for Community Research and Evaluation at The University of Memphis, and other community partners that began in summer 2021. The collaborative seeks to unify efforts to improve outcomes relating to COVID-19 in minority communities living in 11 rural counties of The Delta, which lie along or in proximity to the Mississippi River. Given the unparalleled culture and history of The Delta, it was necessary to get a feel for what vaccine outreach strategies would be most effective in decreasing hesitancy while increasing motivation to receive the vaccine. This study was part of the collaborative’s effort to learn the most effective way forward.

It is critical that populations like those in the Mississippi Delta, who are dealing with intersecting layers of social disadvantage, are not overlooked, both in the national narrative about COVID-19 and in efforts to increase vaccination rates. We shine a light on these important individuals and topics using in-depth interviews of initially vaccine-hesitant Black Americans in the Mississippi Delta and the motivations they weighed in their decision to become vaccinated late.

## Methods

### Study Design

This study was part of a larger effort in which DHA was collecting COVID-19 vaccine information for federal mandates. The goal for this portion of the study was to gain an understanding of how Black residents of The Delta thought about or changed their thinking regarding the virus and vaccines. We utilized in-depth, semi-structured, individual interviews to analyze the meaning-making practices of Black residents in The Delta. An initial discussion guide was developed by the research team, which was practiced internally to extrapolate appropriate questions. Then, a pilot interview was conducted via Zoom to test the quality of the questions. After these exercises, small changes in the wording of the guide were made to improve clarity and to help participants reflect and think about their decision-making process. However, once data collection began, the guide was managed using an iterative process as new themes emerged from the data. The iterative process was aided by team debriefs, which occurred after each interview to monitor the effectiveness of the guide and provided opportunities to add questions surrounding inductive themes of interest to explore in future interviews. The final guide is included in Appendix 2.

### Participants and Recruitment

Participants were recruited using convenience sampling and consisted of employees of DHA. To be eligible for the study, participants were to be comprised of Black employees who were vaccinated after June 2021, *and* residents of the Mississippi Delta. This allowed us to interview people who had access to the vaccination multiple months before deciding to get vaccinated, which revealed how their meaning-making and decision-making processes changed over time. A DHA Human Resources (HR) team member used the sampling information to identify individuals who fit the study criteria and then reached out via email to ask if they would be interested to participate in the study. Upon expressing interest, they were connected with the research team and offered a $50 gift card for participation. We obtained informed consent from all participants to participate in the study and have their respective data published. Ten interviews were completed with nine women and one man who self-identified as Black, with an average age of 34. The sample size was finalized once saturation was reached in the data, and no new topics were generated. See description of participants in Appendix 1.

### Procedures and Data Collection

Interviews occurred face-to-face and were conducted in private, quiet rooms primarily at DHA. One interview occurred at a location that was more convenient for the participant. The interviews were conducted by some members research team in pairs (MA, EM, CB, WJ). Before beginning each interview, informed consent was obtained verbally. The interviewees were informed that everything they said was confidential, participation was completely voluntary, they could leave at any time, and that the recording would be destroyed after transcription. They were also reassured that no one at DHA, including their manager, supervisor, or leadership at the organization would be aware of their participation or anything they said. The interview team used handheld recording devices to capture the recordings. The recordings were downloaded to one of the researcher’s computers (MA) and sent to a third-party company for transcription. Interviews explored participants’ memories of learning about COVID-19, their initial feelings about the vaccines’ release, how the virus and vaccines have impacted their communities, their process of learning about and deciding to get the vaccine, their experiences as vaccinated, and finally, their recommendations about vaccine dissemination to others. Questions were open-ended and allowed the participant to talk about their story and process in the way they wanted. The interviewers used techniques such as unconditional acceptance, active listening, and clarification to promote the authenticity of the data and to avoid bias.

### Data Analysis

Once data were transcribed and returned, all transcriptions were de-identified before being put into MAXQDA software for analysis. The recordings and identified transcripts were deleted from the computer and recording devices. Interviews lasted between 20 and 65 min. Initially, two researchers (MA and CB) free-coded the interviews independently to deductively produce recurring themes. Once free coding was complete, a codebook that included those deductive codes as well as inductive codes based on the literature was developed and finalized. The lead author (AS) then utilized this codebook to reanalyze the data to establish trustworthiness of the data. Conflicting themes were resolved by revisiting the transcripts and through discussion between researchers, adding a layer of intercoder reliability [7]. The researchers reviewed the transcripts for consistency and completed coding. The coding was then developed into major themes that are summarized below.

## Results

Interview data reveal participants’ concerns over the virus and vaccine, reasons for vaccine hesitancy, and the motivations for being late vaccinators. Concerns over COVID-19 and the resulting vaccinations include receiving incomplete and misinformation about each from their social networks and social media; institutional mistrust; and uncertainty regarding side effects or effectiveness of the vaccines. Participants discussed their initial hesitancies, information and relationships that influenced them to change their minds, as well as motivations to receive the vaccine. Among participants’ meaning and decision-making processes was a constant reliance on their own observations, personal conversations, or on religious values or prayer. Interviews resulted in the following themes.

### (Mis)Information-Driven Fear of the Virus

COVID-19 altered the day-to-day way of life for everyone globally—with disparate impacts on poor, rural communities of color. Study participants reported that their (lack of) knowledge about the virus and the people impacted influenced many of their public behaviors. At the onset of the pandemic and the subsequent year after the stay-in-place order of 2020, participants were uneasy because of limited information, inaccuracies of COVID-19 tests, and constantly changing protocols. Participants described the virus’ community impact as heavy-hitting, as all participants knew at least one person, family or friend, who either contracted COVID-19 or died from it. Many participants described the quickness of the virus spreading and the lack of clear information they had, which made hearing about death particularly impactful. Moreover, people were unsure of how to stay safe, maintain their normal lives, and mitigate the fear of getting sick. As Halle described:

…we don’t understand how somethin’ is, is, you know, how it’s all happening. But you hear of people steady, steady dying—you know, from, uh, this thing—and you don’t know the depths of it, you just learn it as you go. ‘Cause people telling you, you gotta wear your mask, you gotta wash your hands, you got to, uh, keep your, your distance and all of that. But you not, I mean, you still having to go to work. You still having, you still having to do certain things to, to keep the flow.

Like others, Halle was overwhelmed by the influx of evolving and even conflicting information. Not knowing enough about contracting COVID-19, how to stay safe while in public, and how to maintain a “normal” way of life caused concern and fear for study participants in ways that caused rifts in community closeness and mental health. Participants saw or heard of increased numbers of people getting sick, making them afraid to leave their homes. Denise called this the “mental load” of dealing with the virus, stating, “[people were] scared. A lot of them hadn’t been vaccinated. And like I say, mentally, it can really—you know—hurt you. It can damage you.” Denise continued to discuss how people were selective about who they would see in person, and that folks were hesitant to send their children back to school because of their fear and reluctance to vaccinate.

Meanwhile, others had no choice but to send students back because of work obligations. Such was the case for Denise and Olivia, who not only have children but also drive a school bus for work. Some participants knew people who could be selective about their contacts, and others who had to return to work. Restricted choices and restricted knowledge caused participants to describe dealing with the virus as putting them “on edge” or hard on one’s mental health. Moreover, the vaccines did not always offer comfort because participants were unsure of the vaccines’ purpose or effectiveness due to conflicting or missing information.

For others, hearing of deaths and witnessing changes in the world made them weary of getting sick or dying. Thinking about these circumstances was exacerbated by other factors as well. As Irene noted:

I started hearing about deaths—people, uh, passing away. And also just going to the grocery store, just as simple as Walmart. There’s no Clorox. There’s no cleaning supplies. There was no tissues. It was like everything was like, 'wow.' Um. I don’t know. At this point, I didn’t know what to do. I didn’t know what to expect. I was afraid, um, I didn't know pretty much too much about the symptoms. It was all about trying to learn what this is as fast as I can to protect my family.

As demonstrated by Halle, Denise, and Irene, participants feared the impact the virus could have on their lives. Additionally, participants were concerned about both the psychological and physical damage the virus could cause to their loved ones and community. Participants were uncertain about safety given a lack of access to resources, and little information to misinformation. While the vaccines should have mitigated such fears, a lack of clear information on the details of the shots caused an added layer of caution.

Several participants reported receiving misinformation from social media, unreliable (mis)information from their social networks, and changing information from media sources. Uncertainties about safety and contracting the virus influenced how participants processed all the information they received about COVID-19, the eventual vaccines, and participants’ hesitancies toward receiving the vaccines. Yet, fear of the virus was not motivation enough for participants to receive the vaccines, as the vaccine was known to carry similar concerns. Further, the disconnect with valid information was compounded by preexisting medical and governmental mistrust.

### Hesitancies Toward the Vaccines: Mistrust, Fear, and Uncertainty

Participants were typically hesitant to receive any COVID-19 vaccines for two main reasons. First, much of the participants’ fear and hesitance about the virus and vaccines were related to their concerns with Black people’s treatment by both medical and political institutions. With a long history of medical mistreatment as unwilling and unknowing experimental subjects and centuries of abuse from the government, Black people are skeptical of such institutions’ intentions. Anti-Black medical malpractice and experimentation result in participants assuming vaccinations will lead to the worst outcome. According to participants, since the initiative to slow the virus’ impact is a joint medical-political venture, the vaccines must be a part of that history. While thinking about vaccines generally, Denise contended, “because we never know what we’re getting. You get what I’m sayin’? Even with the flu shot, we don’t know what we’re getting…” Denise and others were not only cautious about any vaccines because of experimentation, but the limited information available early on about the COVID-19 vaccines too. Similarly, Unique’s thoughts about taking the vaccine reflected Black folks historically being unknowing or nonconsenting test subjects. She asserted:

I felt like, and I’m just gonna be honest—[a] government lab-rat, basically just being a social lab-rat, because we don’t know, um, you know, all the effects of the, uh, vaccine. My biggest fear was getting cancer, um, pretty much any symptom that I’ve never felt before, because of the vaccine. Very, very, terrified. It honestly took me a while to get to vaccine...

Like Unique, some participants worried that the government or medical institutions had nefarious motives, and participants based their thoughts on what they had heard about the effects of the vaccines. Some participants heard of people still getting sick after receiving the vaccine(s)—with mixed messages about the resulting illness (i.e., severity of illness, varied symptoms)—while others read outrageous claims about vaccine reactions or sickness on social media.

Participants reported mixed messages from social media, with some referring to (web)sites as news sources, and most stating that social media spread misinformation and fear. For instance, Keisha held:

So a lot of people promote different things on social media. And it’s been a lot of people, you know, talking against the vaccine– you don’t see too many people on social media saying get vaccinated or a lot of facts or educational things on social media. So this was, I feel like this kind of putting a damper into my community because we all be on social media. And if you constantly see these negative things to where people start to believe those things.

Participants pointed to social media as sources that made them skeptical of the vaccines or caused confusion. Others pointed out that social media posts exaggerate the truth and promote fearmongering. Denise remembered:

I know that social media and the friends on there gonna comment and can rile things up…. Cause you don’t know what to believe. You’ve got some on there might not be telling the truth. They might be scaring you to try to stop you from taking it.

While Denise and others worried that social media might not carry valid information, Wanda was concerned about conspiracy after viewing social media posts. She said:

Well, you know, reading and social media and stuff like this, [people say] ‘they’re getting a chip in it’ and stuff like that. I started thinking to myself, I said, ‘I’m not taking that. They’re not fittin’ to put no chip in me. I’m not gonna take that. They’re not fittin’ to put no chip in me.’

Wanda’s concern is caused by a longstanding ideology that vaccines are more than protection against disease, but also something sinister. All participants reported receiving information from social media, with most questioning the validity of what they had read. Still, such information gave them pause to think. Others were less concerned about conspiracy and more worried with safety generally or as safety directly related to their health. As noted by Unique above, participants were afraid the vaccines would make them sick or still allow them to get sick.

Much of participants’ caution was also due to the speed at which the vaccines were generated. Such thinking led Denise to exclaim:

I think it’s a rush thing, which I still had to come to reality thinking like they can’t take their time because it can get worse. It’s spreading rapidly. You know what I’m saying? So they got to come up with something. And I was like, 'well, what have they tested it on?' ‘Cause, I, you know, and I’m like is it too fast or whatever. But by the negative things… if they don't know nothing about what they experiment on, but they want to stick it in you to give you the virus and don't know what it [might do]– it was just a mind thing. It was just crazy. I was just lost and confused. But like I say, I first- I thought it was made too fast.

Denise thought that the vaccines were necessary because of the spread of the virus, but did not feel safe because of the early lack of understanding about the virus or vaccines—as well as the speed at which the vaccines were created. Other things that concerned her were details about the vaccines’ testing and side effects that she felt were not properly communicated. Ezekiel also pondered the earnestness of the vaccines’ creation and pointed out:

But I think it was more of the thought of, okay, ‘man, we got all these different diseases out here. How can an individual or some form of team come up with a vaccine that can possibly prevent us from getting Coronavirus or, you know, kind of slow it down’—in a sense. You know? I’m just thinking about cancer, I’m thinking about AIDS, I’m thinking about other things too. You see what I’m saying? So- but how can you just all of a sudden say ‘I got this. And I want you guys to put this in your body’?

Like fear of the virus generally, caution about the vaccines’ possible effects—and ineffectiveness—was due to how fast the vaccines were created. Moreover, wariness was related to remaining questions about the virus’ details. Participants were concerned that the quickness of the vaccines’ release meant the shots had questionable and unknown side effects.

Uncertainty about getting sick while vaccinated caused participants to question how an effective vaccine could be developed in such a short amount of time. Participants’ concerns about the vaccines being experimental or developed too fast were connected to their second hesitance—vaccine-related illness. Past reactions to vaccines served to heighten fears in some participants. They were worried that they might have worse side effects after getting the vaccine compared to others. Several pointed to prior reactions to the flu vaccine. They wanted to avoid being sick for even a couple of days, given all their responsibilities. Most indicated they were the primary caretaker for their family. If they were indisposed, their family could suffer for it. Participants reported that when considering the vaccine, they also pondered the necessity given continued recommendations to mask, clean hands, and social distance. Many asked, “why do I have to get vaccinated *and* do all of this stuff?” Such was especially true when they weighed the safety of the vaccine against the preexisting recommendations. When Halle was asked about the safety of the vaccine she stated:

The safety? I feel like they’re still telling us to still wear a mask and still, you know, ‘try to social distance.’ So, I feel like I’m still… even though you have the shot, you still gotta protect yourself, so, it’s not 100%.

During the vaccination decision-making processes, participants weighed the risk of contracting COVID-19 against the perceived risk of the vaccine. Even if they did not think the vaccine was harmful, it certainly was not more effective protection than just general healthy living. Halle recounted her early thinking about the vaccine in the following way:

When I first heard about it, I was, like, ‘I'm not taking nobody’s shot. I don’t feel like I need it. I just need to eat better, take my vitamins, and just take care of myself.’ Because when I first went to get tested for the virus, one of the nurses that was doing it, she actually told me: ‘Well, all you gotta do is just take a certain vitamin.’ So I was, like, ‘Well, maybe that’s pretty much all I need to do, just make sure I’m taking my vitamins – and make sure I’m eating clean and protecting myself.’

Similarly, Ezekiel considered that there must be safer options for staying healthy and safe besides the vaccine. He reasoned:

See, I think it’s more of a thing of ‘I think I can avoid it….’ Um, that is what I believe, you know, if I do the necessary things they say to do to avoid it–wear my mask, wash my hands, sanitize my hands–every time I make contact with somebody or something. Uh, I think I weighed my options when it come to getting that vaccine. See what I’m saying?

Participants like Halle and Ezekiel reasoned that as long as they took other safety precautions like eating well, taking vitamins, and following safety protocols, then there was no need to vaccinate. However, despite their fears of the virus and vaccine or the precautions they took, participants admitted that their minds began to change for several reasons.

### Changing Minds: Motivated to Vaccinate

Participants discussed several reasons why they changed their minds about getting vaccinated. Their motivations included witnessing people’s COVID-19 symptoms without the vaccines versus with them (witnessing social contacts receive the vaccines with minimal symptoms or positive outcomes); a desire to increase their and their family’s safety; returning to work; fear of getting sick; and relying on prayer or faith. Some also began to realize that much of what they had heard was speculation and fear about possible side effects rather than actual complications. As Ezekiel contemplated the claims that vaccines would do harm to the body later, he thought of preexisting vaccines and said, “Just a lot of barbershop talk, man. Look man, you don’t know what that stuff going to do to you in ten years.” Negative talk was remediated by participants’ lived experiences. For instance, Keisha spoke about witnessing the symptoms of the virus with and without the vaccine while working at the hospital. Specifically, she discussed knowing someone whose symptoms were lessened by the vaccine. Keisha noted:

Because just last year before the vaccine, you know, Covid, they was still trying to work up a vaccine and I was working at the hospital during the time. And I seen people that it affected in the worst way. So now my dad just recently had Covid and he’s vaccinated fully. I see the bigger difference. Without having the vaccine and having Covid it’s like I don’t want to – it’s horrible. It’s a horrible sight to see. It’s terrible. But by him being vaccinated and he had Covid it was just like mainly like he had a cold. It was like easy-peasy.

Most participants got the vaccine after coworkers, friends, or family members got it first. While deciding whether to get the COVID-19 vaccine, they observed how others felt after receiving it. Participants had repeated conversations with vaccinated loved ones and coworkers before getting vaccinated. The closeness and openness of these relationships allowed them to feel comfortable asking questions to evaluate the person’s experience. Seeing one or two loved ones have good experiences with the vaccine was pivotal in motivating their decision. Keisha had another contact who received the vaccine and said:

Um, so [co-worker] was the first one to get hers. And, uh, I was like, okay, ‘so how you feel? How was it’? And she actually walked me through the whole thing and how they sign her up, they did her arm, how she felt and everything. And I had asked, I was like, ‘so with your second shot did you feel, you know, sick, fever, chill, or anything?' She was like, just like, ‘some slightly but it wasn’t nothing major or anything.’ And I was like, 'okay'...

Because Keisha had firsthand knowledge from work—patients and co-workers—as well as a loved one receiving the vaccine with positive results, she was motivated to get vaccinated herself.

Similarly, many participants eventually concluded that getting a version of COVID-19 while unvaccinated would be worse for their family, co-workers, and their larger communities than a few days of side effects. On one hand, participants wanted to be safe while working, like Oona whose leadership position requires her to go out into county communities. They also wanted to protect their children and decided being vaccinated themselves could help do that. Specifically, motivation around protection was for themselves and their immediate loved ones, while the general community was only a secondary motivation.

Once participants had received the vaccine, they spent significant time discussing the vaccine with those in their circles and gradually convincing others of its efficacy. Denise talked about her unvaccinated daughter, how she “refresh[ed] her mind every day. Every day, I try to remind her.” Participants felt this repeated communication with trusted individuals was instrumental in their own decision-making, and they also felt it would prove motivational for others.

Beyond direct motivation from contacts to vaccinate, many participants waited to get the vaccine until they felt vulnerable to COVID-19, felt that they were not able to control their exposure, or needed to for social reasons. For example, Olivia recounted her sister delaying vaccination until leaving for college—while Oliva herself waited until she began seeing students on the bus regularly again. Participants felt that in most familial and/or intimate social situations, they could control exposure, but at public-facing jobs, they could not. Lack of ability to control COVID-19 exposure eventually outweighed the perceived risk of the vaccine. Olivia compared herself to under- or unemployed people and discussed her motivation to vaccinate as a labor force necessity. She said, “… myself and my husband, we still have to work. I mean, I still have to come out here every day and transport children to school*.*”

*Spirituality: coping mechanism and vaccine motivator*. Although the interviews did not center on religion or spiritual practices, most of the participants discussed prayer, church (church “family”), or God (the Abrahamic deity) during their conversations. A majority of participants credited God and prayer for their positive experiences and thoughts about COVID-19 and the vaccines. For instance, Ezekiel and Oona credited God with allowing them to never experience the virus. Relatedly, participants stressed their own spirituality and practice of prayer as a part of their decision-making process. Seeking God’s or other spiritual counsel about the decision was almost unanimously part of their decision-making—as they sought a sense of peace about the vaccine. Said differently, faith was a motivating factor to receive the vaccines. Halle, who participated in a prayer phone service with her church, said that she often prayed for her and her community’s safety. Moreover, while thinking about her motivation to vaccinate, Halle said she prayed about it. When asked, “How did you decide what to, what to believe?” she said, “Maybe that prayer [Laughs]. Prayer changes things. And, you know, like I said, I had to be open, I started listening to different things, uh, just opening up more.” Halle reflected on the vaccines and discussed her faith, saying:

I told God, I said, ‘Well, if it's meant for me to get it, God, show me – you gonna, and you gonna give me peace about the whole situation.’ God gave me peace about it, and I ended up just- go ahead and I went ahead and just took it. So, once I got my peace from Him and I felt comfortable enough and I didn't feel like nobody was pushing me – I had peace within myself.

Others said that if they trusted faith, their actions—like getting the vaccines—would be justified. As Oona put it, “I was like, you know we need to get vaccinated, because you know, we trust and believe that…. When you make one step, God makes the rest. So, it’s like, that’s a step for us.” As such, participants’ religious practices helped them rationalize getting vaccinated.

Personal spiritual practices also helped participants look beyond strictly negative thinking, social media posts and comments, or other information sources related to the vaccine. Prayer and faith-guided conversations helped a number of participants realize there might be more benefits to the vaccine than they had previously encountered. Faith also helped participants counter the negative information and conversations they had with others. In response to misinformation about the vaccine having microchips or being a means of social control, Wilma told an acquaintance, “The scientists that created it…. God that- put this knowledge into these, into the scientists to create for us, you know? So it, it must be meant for us to take it.” As such, faith and faith based conversations helped participants make sense of the vaccine. They sought out different perspectives after prayer or spiritual counsel from older individuals. One participant mentioned her husband deciding to get the vaccine after speaking with a mentor she referred to as his “spiritual mother.”

## Discussion

This study examined the experiences and perceptions of Black Mississippi Delta residents surrounding COVID-19 vaccination. All participants were once vaccine-hesitant but were motivated to pursue vaccination, sharing their reasons and decision-making process. The voices in this study are representative of rural minorities who live in communities with multiple layers of social disadvantage, and their perspectives on the decision to vaccinate may resonate with others who felt judged, stereotyped, or unseen during the pandemic. Many of the reasons for hesitancy cited by these participants align with reasons for hesitancy across the nation [7, 12]. For example, apathy toward COVID-19 has been shown to promote vaccine hesitancy [7, 12]. Likewise, distrust of institutions and national politics promotes doubt in vaccine safety and usefulness [18]. For these participants, it was a similar story. Unlike previous studies, participants cited a fear of illness, regardless of vaccination status, and an inability to control exposures as affecting to their hesitance. However, lack of trust for government and healthcare, misinformation, speed of vaccine production, and possible side effects are significant contributors for vaccine hesitancy both nationally and among our participants [7, 12]. Future qualitative research can reveal if there are any uncovered reasons for COVID-19 vaccination that remain.

Our findings support those of Sekimitsu and colleagues (2022), in that vaccination motivators included new understandings of the virus and the vaccines, loved ones receiving the shot, and a desire to live normal, safe lives. Participants engaged in authentic conversations and relationships with individuals who had been vaccinated, which increased their trust in the vaccine. A novel finding is that prayer and personal spiritual practice helped participants embrace positive messaging about the vaccine and consider perspectives they had not previously. As a result, misinformation, close-mindedness, and social media were seen as divisive and dangerous. This pushed participants toward more trustworthy sources of information about the vaccine or to follow positive spirituality. Combined, these factors likely helped participants overcome their institutional mistrust [18]. While the participants of this study were mostly Christian, future research might determine the religious motivations of other faith believers or atheists.

Getting vaccinated to protect themselves, their children, and their communities from COVID-19 was mentioned multiple times by participants. The emphasis on protecting children through adult vaccination appears to diverge from emerging literature on vaccination among women with children at home. Khubchandani and colleagues (2021) found that women with children at home were less likely to get vaccinated compared to women without children at home [26]. Additional research could reveal if race, rurality, or both affect this component. If so, it could be helpful for targeting vaccine education and outreach.

The stories collected in this study offer an opportunity to increase vaccine uptake. Hearing stories of positive experiences with the vaccine, especially from individuals in their socioeconomic circles, was extremely motivating [7, 19]. It could be helpful for workplaces and churches to set up gatherings for groups to talk about their vaccine experiences. This would allow unvaccinated individuals to hear stories of people like themselves having positive vaccine experiences and provide a space for authentic conversations. Additionally, it is not only important for religious leaders to positively talk about the vaccine, but also to encourage their communities to seek out different perspectives through prayer and counsel from trusted health professionals. Finally, most participants did not initially understand the science of how vaccines function in the body. Increased messaging about how the vaccine provides protection should be widely spread.

There are a few additional strengths and limitations with which this study should be weighed. This is one of the first qualitative investigations focusing on rural, Black American perspectives on the COVID-19 vaccines. While a qualitative study such as this does not allow for generalizability to all Black Americans, it does provide important insights into the relevance of racial and place-based vaccine hesitancy. Additionally, the individuals interviewed came from varied class backgrounds and levels of education. They held varying roles (from educator to manager) within the organization (DHA), which helped increase socioeconomic variation within the sample. However, limiting the sample to DHA employees means we were unable to capture the narratives and meaning-making of other disadvantaged residents in The Delta. Our sample’s employment at DHA may have also impacted their motivation to vaccinate. Likewise, the individuals interviewed were overwhelmingly female. Although thematic saturation was achieved, it is possible that conducting more interviews with men could uncover more nuances in this population’s decision-making process around the COVID-19 vaccine. Future studies should continue to get male perspectives, as well as other variations across class, education, and age.

## Conclusion

Many of the reasons for hesitancy cited by these participants align with reasons for hesitancy across the nation. There were some unique motivating factors for vaccination, including personal spiritual practice. Additionally, an emphasis on protecting children through adult vaccination in this group appears to diverge from emerging literature on vaccination among women with children at home. Additional research could reveal if race, rurality, or both affect this component.

## Data Availability

The data that support the findings of this study are not available to individuals or entities outside of the research team due to IRB and funder restrictions.

## Appendix 1 Description of interview participants.

**Table:**

Characteristic	Percent (*N*)	
Female	90% (9)	
Age (yrs) Mean = 34	< 20	0% (0)
	20–30	40% (4)
	31–40	50% (5)
	41–50	10% (1)
	> 50	0% (0)

## Appendix 2. Interview Guide

Hello, my name is [name] and this is [name] and we are working with Delta Health Alliance. [name] will be taking notes during our discussion today. We are part of a team that is looking at data from the RIVER Collaborative grant. The reason for our conversation today is to get some more in-depth information on your experience with getting the COVID-19 vaccine and how you made your decision. We think understanding the reasons people choose to get the vaccine will help healthcare workers and others have more meaningful conversations with individuals they interact with concerning the COVID-19 vaccine. We are attempting to gain understanding by talking to individuals who have recently been vaccinated which is why your specific experience and opinions are important to us. We are not looking for any particular answers because your opinions and experiences are the correct answers. This discussion will last no more than an hour and will consist of open- ended questions. If it is ok, I would like to record our discussion, so that we can most accurately capture your responses. The recording will not be shared and no one in management at Delta Health Alliance or outside of our specific 4-person team will ever hear it. The recording will be deidentified and then transcribed, meaning typed out, for analysis. Do you have any questions at this point? [Pause for response; answer questions if they have any] Any information that might allow someone to know who you are from the recording, like your name or job/position, will be removed from the transcript. The recording will be deleted once we have transcribed it. We are happy to let you know when we have deleted your recording. When you complete the interview you will receive a $50 Walmart gift card.

**Do you have questions about this process?** **Would you like to proceed?** [Get a verbal yes from the individual] [If they say yes, then ask the next question. If they say no, then just reassure them that it is great and remind them that is their right].

**Is it alright if I record the conversation?** [Get a verbal yes from the individual] [If they say yes, then start recording. If they say no, then just reassure them that it is fine, and you will just take notes on the conversation.]

- To start, how did you first hear about COVID-19?
- How has COVID-19 affected your community through the course of this pandemic?
- How about your family/church/friends/coworkers?
- What do you think about the COVID-19 vaccine?
- What about the safety of the vaccine/Effectiveness of the vaccine/Purpose of the vaccine/getting vaccinated?
- What concerned you about the rollout of the vaccine? How have your opinions/feelings changed since, if at all?
- Tell us about how you decided to get the vaccine. How did you make the decision?
- Was there any information you learned that impacted your decision? How did it impact your decision?
- Was there anyone that impacted your decision? How did they impact your decision?
- Did anything happen in your life that impacted your decision? How did it impact your decision?
- How was your experience getting the vaccine?
- What was difficult about your experience? This can be anything leading up to, during, or after your vaccine appointment.
- What was convenient or easy about your experience? This can be anything leading up to, during, or after your vaccine appointment.
- How did you tell your friends about getting vaccinated? How did they respond?
- Family/coworkers?
- Have you recommended the shot to someone? How did you do it?
- Tell us about… what you said/how you said it/what kind of environment were you in
- How would you describe the outcome of the conversation?
- Would you recommend the shot to someone, why or why not? How would you do it?
- Friend/coworker/family member?
- What might be holding people in your social circles back from making the decision that you made?
- Is there anything we didn’t talk about today that you’d like to talk about?

Thank you so much for your contribution today. I know that the information shared today will be so valuable toward increasing understanding of how best to distribute the vaccine and educate about the vaccine. [give the participant the incentive gift card and make sure all documentation for it is complete].
